# Dosage in Vaccine Therapy

**Published:** 1915-05

**Authors:** G. H. Sherman


### DOSAGE IN VACCINE THERAPY.

BY G. H. SHERMAN, M.D.

There is no feature in vaccine therapy that is more puzzling than the question of dosage. Some advocate the use of small doses and report good results, while others insist that large doses are necessary, if the best results are to be obtained. The range of dosage recommended varies anywhere from five to ten millions to several hundred million for the same vaccine. To a casual observer, it may appear that a remedy in which such a variety of dosage is recommended can possess but little virtue. We know, for example, when drugs are given in sufficient doses to produce certain physiologic action, ten or fifty times that dose would be liable to produce disagreeable, if not harmful, effects. Morphine given in ⅛-grain doses will show some physiologic effect, but five times this dose would develop poisonous symptoms.

In this connection, it should be realized that the therapeutic effect of a vaccine is not in any respect a drug action and principles of dosage that would apply to drugs, can not apply to vaccines. Vaccines are given in very small doses. Ten million germs equal approximately 0.000034 gr. and a dose ten times larger 0.00034 gr. still represents an infinitesimal amount.

From the fact that bacterial vaccines are employed in such extremely small doses, it may be inferred that they are extremely toxic in their action when injected into the body; that large doses might have dangerous poisonous effects. Animal experiments will give us some light on this subject. Guinea pigs are as susceptible to biologic remedies as any other animal. We have repeatedly inocculated them with large doses of vaccine without injurious effects. Two guinea pigs were given hypodermically the bacterial contents of a 5 Cc. package of No. 35 (Sherman's) without producing apparent ill-effects.

Comparing the size of a guinea pig to man, this is equivalent to 2,250 times the usual initial dose given for therapeutic purposes. One guinea pig received a hypodermic injection containing the bacterial contents of an 18 Cc. of No. 36 (Sherman's) with no apparent bad effect. This would equal about 8,000 times the usual initial dose given to man. From this, it is clearly evident that bacterial vaccine inoculations are not dangerous.

Vaccine inoculations exert their influence by starting cell activities in the direction of expelling an intruder that possesses the essential characteristics of a dangerous invader. The tissue cells are not aware that the injected germs have been killed and, accordingly, get just as active in their disposal as if a real infection existed at the point of vaccine inoculation. The faculty of self-preservation against infecting organisms of necessity is highly developed, requiring but a small quantity of germs to start the development of specific enzymes, ferments or antibodies for the purpose of disposing of them. When tissue cells have once acquired this faculty of producing specific enzymes, they continue to do so for some time after the infecting organism contained in the vaccine is disposed of and it is for this reason that small doses of vaccine may produce just as good results as larger ones. In fact, experience shows that doses large enough to cause considerable inflammatory reaction at the point of inoculation do not give as good therapeutic results as where moderate reactions take place. It seems that severe reactions have a certain devitalizing influence which hinders the full capacity for enzyme production. The same condition would apply to an infection. We know that virulent infections spread most rapidly. Here the vitality in the intensely inflamed tissue is sufficiently interfered with that their immune producing power is low, whereas under the proper-sized dose of vaccine immune development be obtained to the best advantage.

There are some general rules that have been fairly well worked out which apply to vaccine therapy and allow considerable variation of dosage and yet obtain good results. We find that in extensive acute infections with much fever and other toxic symptoms larger doses are tolerated at shorter intervals with less symptoms of reaction than in subacute and chronic infections. In pneumonia, broncho-pneumonia, puerperal sepsis, erysipelas, etc., a full-sized dose may be given at daily intervals until severe symptoms have subsided while in less severe localized acute or chronic infections, treatment should be started with doses about one-third as large and inoculations made at three to seven-day intervals.—*Bacterial Therapist.*

---

Gov. Ferris has appointed Dr. John S. Hall, of Detroit, a member of the State Board of Dental Examiners for the term ending December 31, 1919. He succeeds Dr. Edgar A. Honey, of Kalamazoo. This is an excellent appointment and it will meet the approval of the entire profession.

